# Team-based learning replaces problem-based learning at a large medical school

**DOI:** 10.1186/s12909-020-02362-4

**Published:** 2020-12-07

**Authors:** Annette Burgess, Jane Bleasel, John Hickson, Ceren Guler, Eszter Kalman, Inam Haq

**Affiliations:** 1 The University of Sydney, Faculty of Medicine and Health, Sydney Medical School, Education Office, The University of Sydney, Edward Ford Building A27, Sydney, NSW 2006 Australia; 2 The University of Sydney, Faculty of Medicine and Health, Sydney Health Professional Education Research Network, The University of Sydney, Sydney, NSW, 2006, Australia; 3 The University of Sydney, Faculty of Medicine and Health, The University of Sydney, Sydney, NSW 2006 Australia

**Keywords:** Team-based learning, Medical curriculum

## Abstract

**Background:**

With increased student numbers in the Sydney Medical Program, and concerns regarding standardisation across cohorts, student satisfaction of the problem-based learning (PBL) model had decreased in recent years. In 2017, Team-based learning (TBL) replaced PBL in Years 1 and 2 of the medical program. This study sought to explore students’ perceptions of their experience of TBL, and to consider resource implications.

**Methods:**

In 2017, Years 1 and 2 medical students (*n* = 625) participated in weekly TBL sessions,

with approximately 60 students per class, consisting of 11 teams of five or six students. Each class was facilitated by a consultant, a basic scientist and a medical registrar. Prior to each class, students were given pre-work, and completed an online Individual Readiness Assurance Test (IRAT). During face-to-face class, students completed the Team Readiness Assurance Test (TRAT), and received feedback with clarification from facilitators, followed by clinical problem-solving activities. Student feedback was collected by questionnaire, using closed and open-ended items. Data were analysed using descriptive statistics and thematic analysis.

**Results:**

In total, 232/275 (84%) Year 1 and 258/350 (74%) Year 2 students responded to the questionnaire. Students found positive aspects of TBL included the small group dynamics, intra- and inter-team discussions, interactions with facilitators, provision of clinical contexts by clinicians, and the readiness assurance process. Suggested improvements included: better alignment of pre-reading tasks, shorter class time, increased opportunity for clinical reasoning, and additional feedback on the mechanistic flowchart. Resource efficiencies were identified, such as a reduction in the number of teaching sessions and required facilitators, and the ability to provide each classroom with clinical expertise.

**Conclusions:**

Our findings demonstrate that TBL, as a replacement for PBL in Years 1 and 2 of the medical curriculum, provided a standardised approach to small group learning on a large scale, and also provided resource efficiencies. Students perceived benefits related to the active learning strategy of TBL that encourage individual learning, consolidation of knowledge, retrieval practice, peer discussion and feedback. However, improvements are needed in terms of better alignment of pre-reading tasks with the TBL patient case, and greater facilitator interaction during the problem-solving activities. Additionally, consideration should be given to reducing class time, and providing TRAT scores.

## Background

Excellence in medical education requires adaptation of the curriculum to meet the changing needs of students. In 1997, Problem based leaning (PBL) was introduced to the Sydney Medical Program (SMP), providing a long-established form of student-centred teaching within the medical curriculum. However, increasing student numbers (from 142 Year 1 students in 1997 to 332 in 2016) and limited teaching resources, rendered this model of teaching unsustainable. With a lack of standardisation across cohorts, extensive face-to-face PBL group meeting time, and limited individual accountability for contribution to group work, student satisfaction with the PBL model had decreased in recent years, and a suitable replacement was sought in Team-based learning (TBL) [1].

First introduced to medical education in 2001, TBL has gained popularity as a resource efficient and student-centred teaching pedagogy. Although TBL remains student centred, it is highly structured, with core design elements, and specific steps [2]. TBL involves multiple small ‘teams’ of five to seven students in one large class room of up to 100 students. Potential advantages of TBL include increased engagement of students in learning, deeper understanding of concepts, and a sense of responsibility towards teammates [1–4]. Additionally, when compared to PBL, TBL maintains the advantages of small group teaching and learning, without requiring such large numbers of teachers [2]. In a recent systematic review on the use of TBL within the health professions, Reimschiesele and colleagues reported that although learner reaction to TBL has been mixed, students generally favour the active learning style of TBL, with multiple opportunities for peer learning, problem solving and feedback [5]. However, there is a gap in literature reporting on the implementation of TBL across entire academic years of the curriculum, involving multiple, large student cohorts. Further to this, we believe that this is one of the first research papers reporting on the large-scale replacement of PBL with TBL across two student cohorts; and providing a direct comparison on the resource and cost implications for such change. Our study provides information and outcomes relevant for educators and curriculum designers working within health professional education.

Following an extensive pilot study in 2016 [1], TBL replaced PBL in the SMP in 2017. This study sought to explore student perceptions of TBL during its first year of iteration in Years 1 and 2 at a large medical school, and to consider resource implications for the change from PBL. Specifically, our research questions were:

What are students’ perception of their experience of TBL?

What are the resource implications when implementing TBL?

## Methods

### Sampling and participants

In 2017, all Year 1 and Year 2 medical students (*n* = 625) medical students participated in the TBL sessions as part of their required teaching activities. Students included Year 1 (*n* = 275) and Year 2 (*n* = 350).

### Content of the TBL sessions

The teaching blocks and number of TBLs completed at the time of data collection (July 2017) are displayed in Table 1. Year 1 teaching blocks included: Orientation and Foundations (3 TBLs), Musculoskeletal sciences (4 TBLs), and Respiratory sciences (4 TBLs). Year 2 teaching blocks included: Neurosciences (6 TBLs), Endocrine, Nutrition, Sexual Health, HIV (6 TBLs), and Renal-Urology (4 TBLs).

**Table 1:** Number of TBLs within each teaching blocks at the time of the study

**Name of the teaching block Number of TBLs**	
**Year 1**	
Foundations block	3
Musculokseletal sciences	4
Respiratory sciences	4
**Year 2**	
Neurological sciences	6
Endocrine and sexual health	6
Renal	4

#### TBL course design

The TBL classes were held approximately once per week across each teaching block on main university campus. Each TBL session was 2.5 h in duration. In Year 1, five TBL classes were run simultaneously. In Year 2, six TBL classes were run simultaneously. Each class consisted of 55 to 60 students, with 11 to 12 student teams per class. Students were allocated to teams to ensure an even distribution of age, gender, and science background. Teams consisted of either five or six students, and teams remained together for the entire year. Each class was facilitated by a team with expertise in the given discipline: one clinician, one basic scientist, and one medical registrar. All student TBL activities (pre-class and class) are summarised in Table 2. We followed Michaelsen and Sweet’s recommendation of three distinct phases to establish the TBL process [6].

**Table 2:** TBL pre-class and class activity schedule

Time	Activity	Explanation of activity
PRE-CLASS ACTIVITIES		
1–2 h	Pre-class reading or pre-recorded lectures	Student pre-preparation was aligned with the TBL of the week.
10 min	Individual Readiness Assurance Test (IRAT) (administered on Blackboard)	Individual knowledge of the pre-reading was assessed by 10 Multiple Choice Questions, using single best answer format, with five options.
CLASS ACTIVITIES		
15 min	Team Readiness Assurance Test (TRAT) (administered via Kuracloud, a cloud-based, content development tool used to deliver interactive lessons)	The same MCQ test was repeated by the students in their teams (TRAT). The test was administered using Kuracloud. One computer or laptop per team was used, with the intent of promoting discussion to establish team consensus. Unlike the 2016 TBL implementation, tutors and students were not told their scores, removing any competition between teams.
25 min	Immediate feedback from the facilitators	The correct answers were given and explained by the facilitators with the use of pre-prepared powerpoint presentations.
105 min	Clinical problem solving activities	Students then worked in their teams on their problem solving activities, using knowledge consolidated through the prior steps.
5 min	Close	

***1) The ‘preparatory phase’****,* where students were allocated compulsory readings or/and pre-recorded lectures prior to class. Additionally, each TBL was aligned with the curriculum course content, so that lectures, laboratory and clinical based teaching were provided prior to the TBL.

**2*) The ‘readiness assurance phase’*,** where the Individual Readiness Assurance Test (IRAT) was implemented as an online pre-class activity, delivered via Blackboard Learn (Learning Management System). The Team Readiness Assurance Test (TRAT) was delivered in class, followed by immediate feedback and clarification by the facilitators.

**3*) The ‘application phase’***, where clinical problem-solving activities were undertaken by students in their teams.

#### Data collection and analysis

### Questionnaire

A questionnaire was distributed to all students following completion of the TBLs listed in Table 1. The questionnaire was anonymous, and distributed by administrative staff, along with a participant information statement (which also explained who was involved in the research), following the last TBL class. The questionnaire included 14 closed items, using a five point Likert-scale, ranging from 1 ‘strongly disagree’, to 5 ‘strongly agree’; and open-ended questions. The questionnaire was adapted from a previously validated questionnaire designed by Thompson and colleagues (2009) [7]. The questions used considered the team processes and dynamics of TBL, for example, *“Team members encouraged one another to express their opinions”.* Additionally, the questions considered the outcomes of the TBL design, for example, *“Completion of the prescribed out-of-class preparation assisted in my learning”.* Quantitative data were analysed using descriptive statistics. Thematic analysis was used to code and categorise qualitative data into themes, building an understanding of students’ expereince throughout the year. A portion of the data was read by the first author and analysed to identify themes. Following negotation of meaning with the third and fourth authors, a coding framework was developed and applied to the full data set [8].

### Ethics approval

Ethics approval was gained from the University of Sydney Human Research Ethics Committee. Approval project number: 2016/136.

## Results

In total, 490/625 (78%) of students responded to the questionnaire, including: 232/275 (84%) Year 1 students and 258/350 (74%) Year 2 students. Of the Year 1 respondents, 119 (51%) were male; 98 (42%) were female; and 15 students (6%) did not respond to this question. Of the Year 2 respondents, 122 (49%) were male; 121 (45%) were female; and 15 (6%) did not respond to this question.

**Fig. 1:**
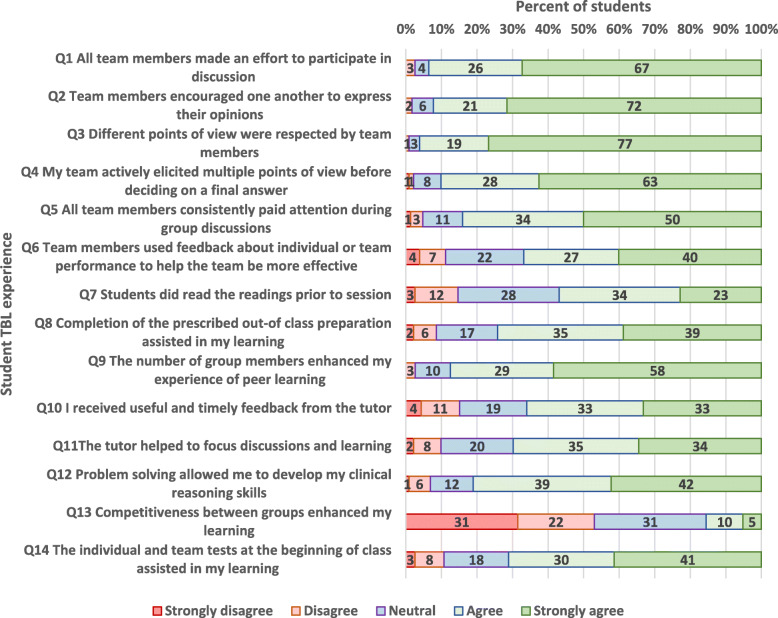
Year 1 student responses to closed items regarding their experience of TBL (*N* = 232)

**Fig. 2:**
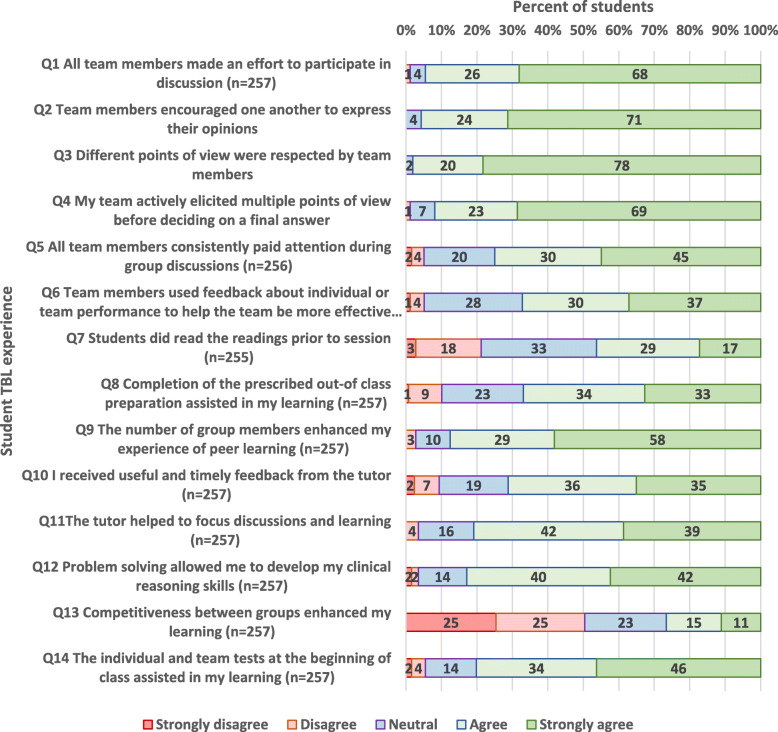
Year 2 student responses to closed items regarding their experience of TBL (*N* = 258)

Student responses to closed items are provided in Fig. 1 (Year 1) and Fig. 2 (Year 2). The distribution of responses across the 5 point Likert scale was similar between the first and second year students. Responses to items 1–5 indicate that the small group dynamics worked well, with most students making an effort to participate, listen, and encourage others in group discussion. Responses to item 7 suggest that assigned pre-readings were not always completed, with only 57% of Year 1 respondents, and 46% of Year 2 respondents agreeing or strongly agreeing with the statement (item 7), *“Students did the pre-readings prior to class”.* Notably, only 17% of Year 1 students, and 27% of Year 2 students agreed or strongly agreed with the statement (item 13), “*Competitiveness between groups enhanced my learning”,* since team scores were not announced.

Students’ responses to open ended questions are illustrated in Tables 3 and 4, categorised as ‘best features of TBL’ and ‘suggestions for improvement/most difficult features of TBL’. Students found the positive aspects of TBL to be the interactive discussion with facilitators who were experts in their field; provision of a clinical context by clinicians; tutors circulating the room to answer questions; the tests (IRAT and TRAT), with the explanations from tutors that followed; peer discussion in small groups (5–6 students); and the interactions with teams in one large room. Suggested improvements included better alignment of pre-reading tasks with the TBL case, increased opportunity for clinical reasoning, and greater feedback on the mechanistic flow charts. Students also suggested a shorter duration of classes (2 hours) and an earlier release of the IRAT to be completed prior to class.

**Table 3:** Students’ perceptions of Team-based learning regarding the ‘best features’

BEST FEATURES OF TBL	
Theme	Examples of student comments
**Presence of Experts**	
Students found it valuable to have content experts as facilitators who could focus the discussion They valued the immediate feedback that was continuously provided by the tutors following the IRAT, and during the clinical problem solving activities	*Exposure to expert tutors … focussed discussion about answers* *Time to fully discuss diseases, access to experts* *Tutors circulating answering questions* *Experts are helpful, integrating the basic sciences and clinical concepts* *Exposure to clinical scenarios and working out links between sign/symptoms and pathophysiology*
**Presence of Clinicians**	
Consultants and Registrars provided a clinical context	*Clinical reasoning discussions with expert tutor* *Experts are available, good clinical reasoning exercise* *Clinical expert tutors, good opportunity to ask them real-world clinical questions*
**Readiness Assurance Process**	
The tests and feedback at the beginning of class helped to focus the session Some students indicated that the Team test (TRAT) motivated them to prepare	*Solving TRAT, answering questions … .explanations given for TRAT questions are detailed* *Reviewing IRAT answers* *TRAT is good, forces you to prepare and do reading*
**Small Groups and discussion with peers** Students found small groups of 5 to 6 students encouraged discussion within groups. Having multiple groups in one room also enriched the learning environment.	*Working as a group is helpful for my learning, learning off others’ expertise* *Meeting other students, accomplishing tasks together is rewarding* *Multiple groups in one room* *Good group, supportive atmosphere*

**Table 4:** Students’ perceptions of Team-based learning regarding the ‘suggested improvements/most difficult features’

SUGGESTED IMPROVEMENTS/MOST DIFFICULT FEATRUES OF TBL	
Theme	Examples of student comments
**Better alignment of the pre-reading content with the TBL case** Students indicated there was a lack of alignment between the assigned pre-readings and the TBL case.	*Questions can include topics not covered in lectures* *Quizzes aren’t always relevant to what is learnt*
**Increased opportunity for clinical reasoning** Some students felt that time was wasted with unnecessary activities, such as rewriting symptoms. They also indicated that too much information was provided, limiting their opportunities for problem solving.	*Rewriting the HPI is not relevant* *Having to rewrite symptoms is redundant* *Too much time spent copying down histories/backgrounds* *Cases are too self-explanatory. Sometimes too hard to correlate BCS with clinical Critical thinking is not* *enough as the diagnosis is already known. Cases are too stereotypical, name of the TBL gives away the diagnosis. The diagnosis should not be so obvious*
**Flow chart explanation** Students felt that further direction, discussion and feedback should be given around the flow-chart activity.	*Expectations of flowchart are not clear* *Lack of targeted questions regarding pathophysiological mechanism*
**Reduction in class time** Students indicated that he 2.5 h given to TBL was too long, which caused them to lose focus	*Too long (lost focus)* *2.5 h is too challenging … .*
**An earlier opening of the online IRAT** The IRAT was only opened the day before the class, and some students had difficulty completing the IRAT in the given timeframe	*IRAT opens too late, don’t have time to do it at home* *IRAT being released too close to TBL not enough time to complete it*

## Discussion

We sought to explore students’ perceptions of their experience in TBL during the first complete implementation of TBL in Years 1 and 2 of the medical curriculum. Results indicate that generally students found their learning experience in TBL to be positive. Students found the positive aspects to be their interactive discussion with experts as facilitators, the presence of clinicians, the tests (IRAT and TRAT) with the explanations from facilitators that followed, peer discussion and problem-solving in small groups, and the inter-team discussion. Suggested improvements included better alignment of pre-reading tasks with the TBL case, increased opportunity for clinical reasoning and interactions with the facilitators; an earlier release of the IRAT; and shorter duration of in-class time. We applied the three phases of TBL: 1) The ‘preparatory phase’, 2) The ‘readiness assurance phase’, and 3) The ‘application phase’, as a framework to discuss our findings. In addition, we consider the resource implications for TBL facilitation.

### The ‘preparatory phase’

Designated preparation for essential knowledge acquisition for TBL was designed to shift the burden of learning content during class. However, students’ compliance with completion of assigned pre-reading and preparation was poorer than expected. Only 57% of Year 1 respondents, and 46% of Year 2 respondents agreed or strongly agreed with the statement (item 7) *“Students did the pre-readings prior to class”.* This is a reduction from the 68% of students who were in agreement with this statement in the 2016 pilot study [1]. Failure to prepare impacts on the teams’ learning and performance, and is an important area to address [9]. The recommended practice in TBL is to announce the TRAT scores to the class [10], however, this step was not included in the 2017 TBL implementation. Perhaps the lack of student preparation indicates that the announcement of team scores may have helped motivate students to complete pre-readings and attend class better prepared to engage and contribute to their teams. Additionally, qualitative feedback from students indicated that there was a lack of alignment of the test with the pre-reading material. It is recommended that preparation material be identified after the multiple choice questions have been written to ensure relevance of the material, and completion of assigned preparation [2].

### The ‘readiness assurance phase’

The tests (IRAT and TRAT) were well received by students, with the majority of students (71% of Year 1 students, and 80% of Year 2 students) agreeing or strongly agreeing that the “*individual and team tests assisted in my learning”.* In order to reduce in-class time, the IRAT was held prior to class, made available to students 1 day prior. However, a number of students requested that the IRAT be released earlier. Literature suggests that taking the IRAT online prior to class adds value to learning [11]. Additionally, there is evidence to suggest that repeated testing of students encourages retrieval of new knowledge, and helps in recall of knowledge at a later date [12]. Opportunities for critical reflection are needed to allow students to make judgements on required modification to their existing knowledge [13]. Our results suggest that reflection occurred when students compared their understanding to that of their team members during the TRAT, through discussion to agree on an answer. Notably, 94% of Year 1 students, and 95% of Year 2 students strongly agreed or agreed that *“all team members made an effort to participate in discussion”*, suggesting that the small group size enhanced team dynamics. Unlike the TBL format in 2016, the TRAT results were not released to the class. Therefore, it is not surprising that only 17% of Year 1 students, and 27% of Year 2 students agreed or strongly agreed with the statement (item 13), “*Competitiveness between groups enhanced my learning”,* compared to 69% (agreeing or strongly agreeing) in the pilot study [1]. However, current TBL literature and research suggests that friendly competition is an important component to the TBL process [1–4], indicating that this is an element of the TBL implementation requiring further consideration.

### The ‘application phase’

The quality of clinical problem-solving activities plays a key role in engaging students in small group collaboration [14]. Importantly, students generally felt satisfied with the problem-solving activities within TBL, with 81% of Year 1 students, and 83% of Year 2 students strongly agreeing or agreeing that *“problem solving allowed me to develop my clinical reasoning skills”*. Students appreciated the expertise of facilitators, and the clinical context provided by consultants and registrars. The importance of providing clinical relevance to medical teaching is frequently highlighted in medical education literature [15]. However, students indicated a need for increased direction and discussion regarding the pathophysiology flowchart. Literature suggests that immediate feedback may assist student groups to work more independently throughout the problem-solving activities [16, 17]. Further to this, only 66% of Year 1 students and 71% of Year 2 students agreed or strongly agreed with the statement (item 10), *“I received useful and timely feedback from the tutor”.* In our 2016 pilot study of TBL, 80% of students had agreed or strongly agreed with this statement [1], which may reflect the extensive teaching experience of the 2016 facilitators, who were all senior consultants.

### Resource implications

Although the key reasons for implementing TBL were to provide greater standardisation in teaching and an improved educational experience to students, it is also important to consider the resource efficiencies of TBL compared with PBL. In 2017, there were 625 Years 1 and 2 medical students enrolled in the medical program. Using the TBL format, with approximately 60 students per class, we implemented 11 TBL classes per week, requiring a total of 33 facilitators (3 facilitators per TBL class). The previous PBL format, with 10 students per PBL, required 62 facilitators (one facilitator per PBL group) each week. By implementing TBL, we have reduced our facilitator requirements by approximately half, and at the same time provided clinical expertise in each classroom. This has obvious cost saving implications. It should be noted, however, that the preparation required for each TBL module was time consuming, and required academic expertise. Tasks include preparation of multiple choice questions and answers, development of patient cases and clinical problem-solving activities, and Powerpoint slide presentations to guide feedback and clarification of concepts.

### Study limitations

Data collection occurred after completion of three blocks of Year 1 teaching, and three blocks of Year 2 teaching. It is possible that students’ perceptions at that point in time differed from their perceptions at the end of the academic year. The findings from our study may not be generalisable to other universities. A limitation is that although the questions within our questionnaire were based on a validated questionnaire designed to measure the quality of team processes [7], our questionnaire itself was not validated prior to use.

## Conclusion

Our findings demonstrate that TBL, as a replacement for PBL in Years 1 and 2 of the medical curriculum, provided a standardised approach to small group learning on a large scale, and also provided resource efficiencies. Interactions and discussion with experts as facilitators, the presence of clinicians in every class; the repeated testing (IRAT and TRAT) with feedback and explanation; small group peer discussion and problem-solving were all aspects of TBL experience that the students found positive. However, improvements are needed in terms of better alignment of pre-reading tasks with the IRAT, and greater facilitator interaction during the problem-solving activities. Additionally, consideration should be given to reducing class time, an earlier release of the IRAT, and provision of TRAT scores to the class.

## Data Availability

Datasets supporting the conclusions of this article are included within the article. Additional data at the level of individual students is not available as per confidentiality agreements approved by the Human Research Ethics Committee, University of Sydney.

## Abbreviations

TBL: Team-based learning
PBL: Problem based learning
IRAT: Individual readiness assurance test
TRAT: Team readiness assurance test
SMP: Sydney medical program
